# Using e-Delphi to formulate and appraise the guidelines for women’s health concerns at a coal mine: A case study

**DOI:** 10.4102/curationis.v41i1.1934

**Published:** 2018-10-04

**Authors:** Princess N. Msibi, Ramadimetja Mogale, Maretha de Waal, Nomathemba Ngcobo

**Affiliations:** 1 Department of Nursing Science, University of Pretoria, South Africa

## Abstract

**Background:**

E-Delphi is an online method widely used in health and social research to strengthen decision-making processes and reach consensus on developing guidelines for health services.

**Objectives:**

The e-Delphi technique was designed to formulate and appraise a set of criterion-referenced guidelines for women’s health concerns of mineworkers at a selected coal mine in Mpumalanga, South Africa.

**Method:**

The University Learning Management System’s discussion forum was customised to suit the characteristics of e-Delphi as the second phase of a primary study on the formulation of guidelines for women’s health concerns. Six purposively sampled experts with extensive experience in Occupational Health and in Women’s Health participated. Online engagements on the formulation and appraisal of the guidelines for women’s health concerns took 7 weeks, divided into four phases as preparatory, exploratory, consensus and refinement. From the experts’ inputs, guidelines were drafted. Experts were invited to evaluate the guidelines by using a 7-point Likert scale with AGREE II criteria. Consensus was reached in two e-rounds.

**Results:**

Eight guidelines were formulated, appraised and adopted as: change management, control of hazardous environments, suitable psychosocial working environment, provision of health care service, uphold human dignity and adherence to human rights, effective measures for safety participation compliance, accessible, available and relevant on-site health care services and hope and resilience. Each guideline has rationale, operational strategies and anticipated outcomes.

**Conclusion:**

E-Delphi platform used various tools to deliberate on the process of guidelines formulation and appraisal. The platform was convenient for the experts’ participation in the discussion at any time and anywhere.

## Introduction

Techniques such as traditional Delphi, nominal group discussions and, recently, e-Delphi are used to reach consensus on issues related to nursing and health practices (Graefe & Armstrong 2010; WHO 2010). Unlike the traditional Delphi technique, which is commonly used as a formal consensus method with two or more rounds of face-to-face interactions, the e-Delphi assembles ideas online with experts communicating and engaging with each other at their own time at their vicinities and either in an asynchronous or anonymous manner (Thangaratinam & Redman 2005). The e-Delphi studies create opportunities for researchers, including nurse researchers, to conduct research nationally and internationally, provided the researchers carefully consider such designs and methods as part of data collection. E-research methodologies, such as the e-Delphi technique, have yet to undergo significant critical discussion in respect of possible risk of bias and applicability (Toronto 2017), as not all evidence is the same. Evidence in research is described in accordance with how it is situated in the evidence pyramid (Davidson & Candy 2016). Evidence pyramids represent a hierarchy of internal validity (risk of bias) or both internal and external validity (applicability) (Murad et al. 2016) of knowledge.

This article has four sections. Firstly, the introduction discusses why the lower sources of evidence in the hierarchy are preferred in nursing practice. Secondly, the article presents information about the e-Delphi as a technique in reaching consensus. Thirdly, the article expounds on the entire process that was followed to design an e-Delphi platform through the University Learning Management System (LMS) and the processes that were followed through online engagements to formulate and appraise the guidelines. The article ends with two examples of what the formulated and appraised guidelines look like.

The evidence from expert committee reports or opinions and/or clinical experience of respected authorities falls within Level IV in the evidence pyramid. Most experts agree that the higher up the hierarchy the study design is positioned, the more rigorous the methodology (Davidson & Candy 2016). The study design can minimise the effect of bias on the results of the study. Study design, together with methodological limitations of a study, imprecision, inconsistency and indirectness are additional factors that can affect the quality of evidence (Van der Linde et al. 2005:693). That said, lower sources of evidence in the hierarchy are least preferred in nursing practice because they require extensive expertise and time for identification, appraisal and application. In nursing, evidence-based nursing is a way of providing nursing care that is guided by the integration of the best available scientific knowledge together with nursing expertise.

Nursing researchers interested in harnessing expert knowledge on a broad range of health and well-being topics are increasingly using Delphi as a means of capturing collective opinion (Brüggen 2009). The method has been adopted across a wide range of health, social care and well-being studies related to policy, clinical practice, planning and evaluation (Brüggen 2009). Additionally, Delphi techniques are also used to reach consensus in the development of guidelines (Lindell & Demi 2018). While the use of Delphi is increasing in health and well-being research, less attention has been given to the methodological underpinnings of the Delphi method, especially the e-Delphi technique as a newly emerging e-research method for reaching consensus. Likewise, Internet-based research is growing rapidly in popularity; however, it has yet to undergo critical methodological review (Toronto 2017). Despite efforts to capitalise on the potential of information technology for research and practice in health, inadequate attention has been paid to how knowledge from these new endeavours can be validated. This is because some e-research methods are commonly used to reach a consensus in the absence of research evidence (Toronto 2017).

## e-Delphi

Delphi is a technique used to achieve a common viewpoint from experts using questionnaires to gather information of interest (Lindell & Demi 2018). This technique has been extensively used within health and social research to strengthen decision-making processes and reach consensus. According to Green (2014), e-Delphi is defined as a method for structuring a group communication process so that the process is effective in allowing a group of individuals, as a whole, to deal with multiple challenges. The e-Delphi method allows experts to communicate and engage with each other online in their own time in their vicinities to solve problems until consensus is reached. According to Bardhan, Ngeru & Pitts (2012), the e-Delphi technique in this era is important in evidence-based research as it enables participants to post their opinions and accrue their ideas online.

With the use of the e-Delphi technique, experts participate in different periods of time, at their own pace (Meshkat et al. 2014). Other researchers (Mamaqi, Miguel & Olave 2010) have agreed recently that the use of e-Delphi is ideal; for one thing, it is anonymous because neither the researcher nor the experts was physically present, which might influence the communication and lead to prejudice. Such bias may further lead to insufficient and incorrect data collection and lack of evidence, which could in turn lead to inability to reach consensus in the guideline formulation. In the e-Delphi process, reaching consensus by experts cannot be resolved in a once-off discussion. As an iterative process, e-Delphi involves a chance for initial feedback, collation of feedback and distribution of collated feedback to participants for further review. Depending on the number of research questions and available time to reach consensus, the e-Delphi process includes three rounds, to prevent exhaustion and attenuation (Green 2014). Furthermore, e-Delphi as a method strengthens the diversified co-learning through a shifted paradigm in a guideline formulation arena. In addition, some of the common biases that usually occur in a face-to-face group process are removed (Eubank et al. 2016).

## Methods

### Designing the e-Delphi

An e-Delphi platform was designed as the second phase of a primary study on the formulation of guidelines for the health concerns of the women mineworkers at the selected coal mine.

### Context of the study

**FIGURE 1:**
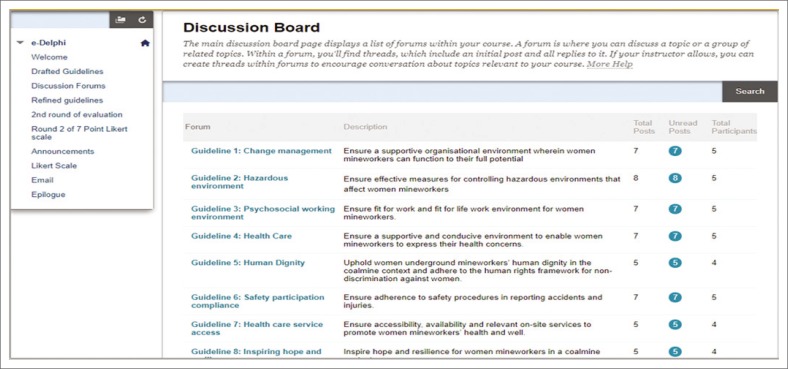
Some features of the e-Delphi.

A discussion board from the LMS was customised to meet the requirements of an e-Delphi (Chou 2002). According to Chou (2002) these requirements are the two online interfaces for the project leader and the experts. The interface for the project leader is used for the development and sending of questionnaires to the experts. The interface for the experts is to respond and give inputs to the project leader. Additionally, the platform should allow for other forms of multimedia communication, for example self-recorded podcasts in this case. Of importance, the platform should permit the project leader to totalise and analyse the responses of the experts easily (Brüggen 2009). This e-Delphi had the following features: login page, contact page, landing page, discussion forum, file upload area, communication tools (announcements and email), user guide (for external experts on how to access the LMS and use the system and tools), YouTube link for podcasts (because of the limited space in the LMS to upload large file sizes, podcasts were uploaded to YouTube as unlisted - not searchable - videos, then a link was made available to the LMS) and a Likert scale. See Figure 1 for some of the features of the e-Delphi.

### Sampling criteria

E-Delphi employs ‘experts’ as panel members (Taylor et al. 2016:2). The experts who participated in this study were selected through purposive sampling. Only 6 experts out of the initial 12 provided the written informed consent to be part of the online discussions, though. A formal written request in accordance with the university’s policy was sent to the LMS administrator to provide the experts with online access, as they were not affiliated to the university. Not only were the experts from the mining and factory industries, but some were from municipality and government entities such as Transnet and Eskom. All of them had extensive experience in Occupational Health and some in Women’s Health. To enhance the rigour of the guidelines, an external expert was also selected purposively for expertise in guideline development within Occupational Health and Safety. See Table 1 for the characterisation of the experts.

**TABLE 1:** Characterisation of the experts.

Expert	Occupation	Years of experience	Occupational health qualification and experience
1	Registered nurse, Occupational Health	21 years as Occupational Health practitioner	B. Tech in Occupational Health Nursing
2	Registered nurse, Occupational Health practitioner	18 years	B. Tech in Occupational Health Nursing
3	Registered nurse, Occupational Health practitioner	20 years	B. Tech in Occupational Health Nursing
4	Professor, researcher and an Occupational Health specialist	15 years	PhD qualification in Nursing and Occupational Health
5	Registered nurse with PhD qualification	20 years in Women’s Health	PhD qualification in Nursing and research
6	Professor in Sociology with PhD qualification	20 years in Women Studies	PhD qualification in Sociology and research

Five of the experts who participated in the e-Delphi were registered nurses, three had a bachelor’s of technology in Occupational Health Nursing, while the other three were PhD prepared scholars. The experts’ years of experience ranged from 15 to 25 years in Occupational Health and Women’s Health and Issues.

## Online engagements

The online engagements were collected through the discussion forums. These forums were used asynchronously at the users’ own time and pace, with the duration of rounds clearly highlighted for experts. The forums ran for a period of 7 weeks in succession. The 7 weeks were divided into four phases: preparatory, exploration, consensus, with refinement as the last phase.

### Phase 1: Preparatory phase

In this phase both the instructional designer and the system administrator of the LMS played an important role in the preparation and then design of the e-Delphi. As a registered student, the researcher had free access to the LMS. The experts were provided with individual official login details by the system administrator. The user manual on the steps to log in on the e-Delphi platform was developed by an instructional designer. This user manual included the use of particular tools of the LMS. The researcher was trained as the moderator on how to use the platform and self-recording of the podcasts by the department’s instructional designer. The first podcast outlined the processes that the experts were to follow during the entire period of the e-Delphi. The researcher tapped the socio-economic model (SEM) to identify eight provisional statements to be considered in the formulation of the guidelines from the findings of the main study. This phase took 1 week.

### Phase 2: Exploratory phase

The aim of this phase was to brainstorm the purpose and scope of the guidelines. The provisional statements, which were optimised with the self-recorded podcasts, were posted on the e-Delphi platform. Hands-on support was provided to the experts through email messages and telephonic support throughout the project period. This phase lasted for 4 weeks. The experts were required to provide inputs and suggestions on the content of the statements, which were to be guidelines for the underground women’s health concerns in the selected coal mine. Following are some of the examples of such inputs and comments on the statements (see Box 1).

Box 1 Expert inputs and suggestions.

**Expert 3**

‘Change is always necessary but never easy. When change is managed by including all parties it goes a long way to inform, increase confidence and ease the ability of management to lead.’

‘Policies and procedures go a long way in ensuring compliance of most actions in the workplace. Testing ideas before implementation strive to have an informed staff. Problems need to be tackled head on. As the mining industry comes from a long history of male domination, we should educate and encourage people to change. Identify the change and take active and deliberate attempts to embrace the change.’

**Expert 1**

‘Women [*in mining*] work in a hazardous environment, they will need to ensure that they are wearing the correct PPE. The PPE is the overall, hard hat, goggles if needed, harnesses, ropes, safety boots, and so forth. All these PPE, are heavy, which is another weight, adding on the heavy tasks that need to be performed.’

**Expert 2**

‘It is really a difficult, non-conducive environment to allow a woman to work in such conditions, mainly because of the physical strain that would allow one to be exposed to. … Every month a woman go through menstrual cycle whereby she will need to refresh herself often, by changing a sanitary pad, or tampon. It is a fact that there is no built ablutions underground which exposes a woman to an unhealthy working environment which is totally contrary to what the Acts emphasise.’

PPE, personal protective equipment.

### Phase 3: Consensus phase

From the inputs and comments of the experts in the exploration phase (Box 1) via the e-Delphi the researcher drafted eight guidelines. A seven-point Likert scale using the AGREE II tool was developed. The AGREE II tool evaluates the methodological rigour and transparency in guideline formulation (MacDermid et al. 2005). The tool has six domains, with Domain 1 as the scope and purpose, Domain 2 being stakeholder involvement, Domain 3 rigour of development, Domain 4 clarity of presentation, Domain 5 applicability and Domain 6 about editorial independence (MacDermid et al. 2005). The overall evaluation required the AGREE II user to make a judgement as to the quality of the guideline, taking into account the appraisal items on a seven-point Likert scale (Brouwers et al. 2010). A score of 1 is given when no information was relevant to the item or if the concept was very poorly reported. A score of 7 was given if the quality of reporting was exceptional and where the full criteria and considerations articulated had been met. A score between 2 and 6 was assigned when the reporting of the item did not meet the full criteria or considerations. In addition, a score was assigned depending on the completeness and quality of reporting (MacDermid et al. 2005). The score increased when more criteria were met, and the aspect or determinant was addressed (MacDermid et al. 2005).

**FIGURE 2:**
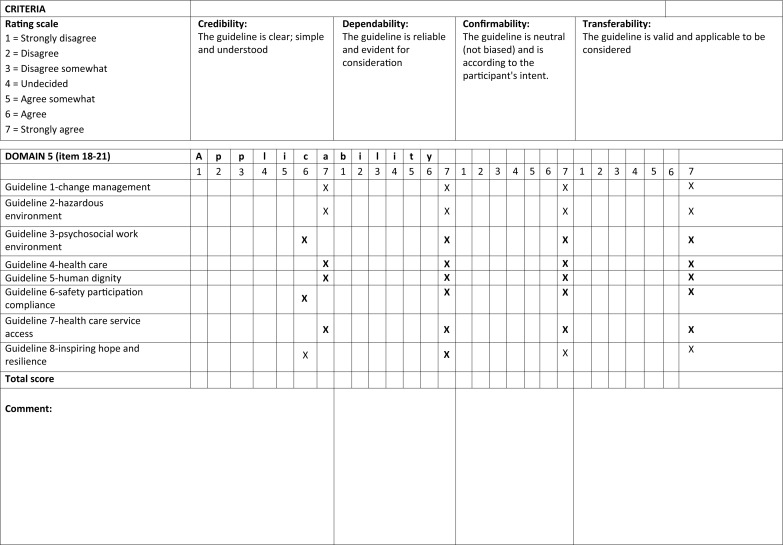
An example of the rated scale from the e-round.

Likewise, the process was followed in this e-Delphi technique and only two e-rounds were used to reach consensus. In the first e-round, the experts evaluated the guidelines with different scorings ranging from 4 to 7 together with the comments and concerns. Those with 7 as a score were not sent again to the experts. Refer to Figure 2 as an example of the rated scale from the e-round. The project leader incorporated the comments in the guidelines and posted them for evaluation for the second round. On the second e-round, the experts evaluated the refined guidelines and gave a score of 7, and consensus was reached.

### Phase 4: Refinement of the guidelines

With the drafted guidelines from the two e-rounds, the project leader solicited advice from an external expert. The external expert provided constructive comments on how to rearrange and reconstruct the guidelines. From the external expert’s comments, the project leader refined the drafted guidelines, finalised and presented them to the experts, who then adopted the guidelines.

## Measures to ensure trustworthiness

The researcher ensured credibility by the ongoing iteration and feedback from and to the experts, which according to Engles and Kennedy (2007) is viewed as member checks. To achieve dependability, the researcher included a range of experts (Cornick 2006) from mining and industry as well as from the municipality. Confirmability was assessed by maintaining a detailed description of the e-Delphi collection and analysis processes. Transferability was established using verification of the applicability of e-Delphi findings.

### Ethical considerations

The study obtained ethical clearance from the Faculty of Health Sciences ethics committee. (Ethics Reference number 206/2015). During the selection phase, the information leaflet which had the relevant information about the e-Delphi process and informed consent was emailed to the experts as indicated under the sampling criteria. Participation in the e-Delphi was voluntary, anonymous and confidential (Thangaratinam & Redman 2005).

## Results and discussions

Eight guidelines were formulated to address women’s health concerns in the selected coal mine. These were: change management and supportive organisational environment, control of hazardous environments, creation of effective and suitable psychosocial working environment and provision of health care service where women can express their health concerns upholding of human dignity and adherence to a human rights framework, effective measures for safety participation compliance, accessible, available and relevant on-site health care services, hope and resilience. Each guideline has a rationale, operational strategies and anticipated outcomes, as shown in the following two examples (see Box 2 and Box 3).

Box 2 Creating effective measures to enforce change management and a supportive organisational environment.

According to Rijal (2009:132) and Szamosi and Duxbury (2002), change management is an integral part of most organisations, as it is the way people, teams and companies change, using different methods to redirect utilisation of resources, allocation of budgets, business processes or any other plan of work that will transform the organisation.

**Rationale**

To promote change while encouraging the uptake of change among the employees, management should drive for the values that underpin change management while improving communication at the workplace.

**Operational strategies**

Policy changes may have a positive effect if all the mineworkers, including women, are part of the change.

**Anticipated outcomes**

Mine management are expected to commit and adhere to ethical responsibilities with no exceptions between women and men mineworkers.

Box 3 Create effective measures for controlling hazardous environments that affect women mineworkers’ health.

The *Basic Conditions of Employment Act* (No. 75 of 1997) and the Chemical Substances Regulations (1995) define a hazardous environment in mining as an area that can cause harm or damage to humans, property and/or a place.

**Rationale**

Despite coal being a national asset and a primary source of energy in South Africa, coal mining endangers human life because of the release of noxious and poisonous gases.

**Operational strategy**

Mineworkers should wear PPE throughout the shift when on duty. Mine management should enforce wearing of such while the occupational nurse should conduct employee risk assessment regularly.

**Anticipated outcome**

If correct PPE is provided and enforced, women’s health will not adversely affected.

PPE, personal protective equipment.

## Implications and conclusions

The article highlighted how positive deviance was used to leverage the LMS to design an e-Delphi. The process followed to design the platform may offer scholars unique insights on how to incorporate such techniques into future studies. Nurses are encouraged to utilise their knowledge and skills to creatively generate and develop innovative ways of conducting research as potent technologies are being developed with the potential to transform society.

The e-Delphi technique has proved to be a resourceful, systematic and cost-effective method of accelerating knowledge generation in nursing sciences and research. The platform provided a space to reach consensus on the formulation and appraisal of the guidelines. Various e-tools were used to deliberate on the process of formulation and appraisal of these guidelines. The platform was convenient for the expert as they could participate in the discussion any time and anywhere.
